# Transmission dynamics reveal the impracticality of COVID-19 herd immunity strategies

**DOI:** 10.1073/pnas.2008087117

**Published:** 2020-09-22

**Authors:** Tobias S. Brett, Pejman Rohani

**Affiliations:** ^a^Odum School of Ecology, University of Georgia, Athens, GA, 30602;; ^b^Center for the Ecology of Infectious Diseases, University of Georgia, Athens, GA, 30602;; ^c^Department of Infectious Diseases, University of Georgia, Athens, GA, 30602

**Keywords:** infectious diseases, mathematical modeling, dynamical systems

## Abstract

The rapid growth rate of COVID-19 continues to threaten to overwhelm healthcare systems in multiple countries. In response, severely affected countries have had to impose a range of public health strategies achieved via nonpharmaceutical interventions. Broadly, these strategies have fallen into two categories: 1) “mitigation,” which aims to achieve herd immunity by allowing the severe acute respiratory syndrome coronavirus 2 (SARS-CoV-2) virus to spread through the population while mitigating disease burden, and 2) “suppression,” aiming to drastically reduce SARS-CoV-2 transmission rates and halt endogenous transmission in the target population. Using an age-structured transmission model, parameterized to simulate SARS-CoV-2 transmission in the United Kingdom, we assessed the long-term prospects of success using both of these approaches. We simulated a range of different nonpharmaceutical intervention scenarios incorporating social distancing applied to differing age groups. Our modeling confirmed that suppression of SARS-CoV-2 transmission is possible with plausible levels of social distancing over a period of months, consistent with observed trends. Notably, our modeling did not support achieving herd immunity as a practical objective, requiring an unlikely balancing of multiple poorly defined forces. Specifically, we found that 1) social distancing must initially reduce the transmission rate to within a narrow range, 2) to compensate for susceptible depletion, the extent of social distancing must be adaptive over time in a precise yet unfeasible way, and 3) social distancing must be maintained for an extended period to ensure the healthcare system is not overwhelmed.

## Significance

Confronted with escalating COVID-19 outbreaks, countries at the leading edge of the pandemic have had to resort to imposing drastic social distancing measures which have serious societal and economic repercussions. Establishing herd immunity in a population by allowing the epidemic to spread, while mitigating the negative health impacts of COVID-19, presents a tantalizing resolution to the crisis. Our study simulating SARS-CoV-2 spread in the United Kingdom finds that achieving herd immunity without overwhelming hospital capacity leaves little room for error. Intervention levels must be carefully manipulated in an adaptive manner for an extended period, despite acute sensitivity to poorly quantified epidemiological factors. Such fine-tuning of social distancing renders this strategy impractical.

Caused by a novel coronavirus, severe acute respiratory syndrome coronavirus 2 (SARS-CoV-2) (1), COVID-19 is an infectious disease capable of severe respiratory illness and death (2). Since its identification in Wuhan, China, COVID-19 has become an on-going and rapidly expanding global pandemic that is causing substantial mortality and healthcare system strain in multiple countries (3). While older individuals and those with underlying conditions are most at risk (4), infection has been seen across age groups (5, 6). Worryingly, detection of viral loads in the upper respiratory tract suggests potential for presymptomatic and ogliosymptomatic transmission (7–9). Due to the absence of a vaccine, current attempts at controlling SARS-CoV-2 spread are focused on social measures that reduce rates of viral transmission: social distancing (a generalized reduction of contact rates between individuals in the population) and self-isolation by symptomatic individuals (10).

Broadly speaking, two distinct approaches to controlling the spread of SARS-CoV-2 have received much attention. The first aims to suppress transmission in the target population (referred to hereafter as “suppression”) (10). Under this objective, control measures reduce viral transmission to such a degree that sustained endogenous transmission is no longer possible. By maintaining control measures in place for a sufficient period of time, the virus will be eliminated in the focal population. The focus will then shift to preventing subsequent reintroduction to prevent resurgence. The second approach aims to manage or mitigate the negative health impacts (referred to hereafter as “mitigation”) (10). While suppression aims to ultimately halt local transmission, mitigation aims to reduce the growth rate of the epidemic to ensure disease burden does not overwhelm healthcare systems (3). By reducing (rather than halting) transmission, this strategy allows the susceptible pool to diminish, with the population potentially able to achieve herd immunity (whereby sustained local transmission is impossible, even without social distancing) (11). In practice, both approaches require the rollout of the same types of control measures (social distancing and self-isolation), although the necessary intensities and durations vary.

At the time of writing, trends in incidence data suggest multiple countries, including China, South Korea, Spain, and Italy, have successfully implemented suppression strategies (12). Sweden is the poster child for mitigation strategies, and appears to be aiming for herd immunity (13). Meanwhile, other countries continue to experience sustained transmission, and strategic intention is less clear [including the United States (14) and United Kingdom (15)]. The severe economic costs and acute societal pressures associated with social distancing measures have led to a push for their relaxation (10). Given the potentially long wait until a vaccine is available, the UK government appears to have considered following Sweden’s example and attempt to achieve herd immunity in the country (16).

The consequences of failure to either adequately mitigate or suppress COVID-19 are potentially catastrophic. Due to the many uncertainties surrounding SARS-CoV-2 transmission, evolution, and immunity, public health decision makers are presented with an unenviable task. To help inform control policies under uncertainty, mathematical modeling can assist in evaluating the viability of mitigation and suppression as objectives (17), by simulating the impacts of control strategies on viral transmission, hospital burden, fatalities, and population-level immunity.

Recent studies have modeled impacts of both mitigation and suppression strategies, including for China (18), low-income countries (19), and the United Kingdom (20, 21). Crucially, we are not aware of any systematic studies that focus on 1) walking the tightrope of achieving herd immunity without overburdening healthcare systems and 2) the control effort (e.g., reduction in contacts) required for successful mitigation relative to suppression. These two knowledge gaps motivate our study. We use an age-stratified disease transmission model, taking the United Kingdom as an example, to simulate SARS-CoV-2 spread controlled by individual self-isolation and widespread social distancing. We simulated various levels of self-isolation effectiveness and three distinct types of social-distancing measures: 1) school (including university) closures, 2) work and social place closures, and 3) effective isolation by older individuals.

Our modeling confirms that suppression is possible with plausible levels of social distancing and self-isolation, consistent with experience in multiple countries. Our research does not, however, support attempting to mitigate COVID-19 with the aim of building herd immunity. Achieving herd immunity while simultaneously maintaining hospital burden at manageable levels requires adaptive fine-tuning of mitigation efforts, in the face of imperfect epidemiological intelligence—something that is impractical.

## Results

**Fig. 1.**
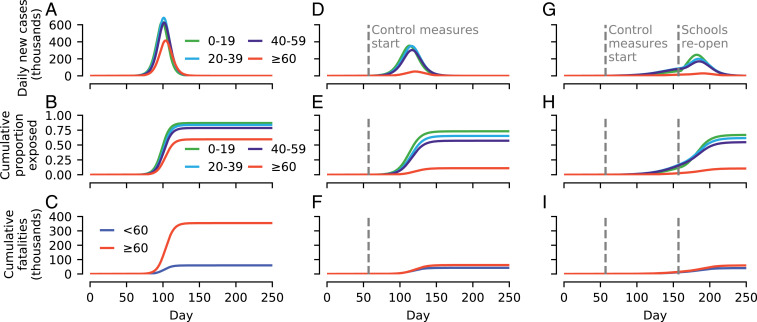
Simulated examples of SARS-CoV-2 spread in the United Kingdom using an age-structured Susceptible Exposed Infectious Recovered (SEIR) model under different control scenarios. (*A*) Daily new cases assuming no intervention measures enacted. (*B*) Cumulative proportion of the population exposed to SARS-CoV-2 over the course of the epidemic. (*C*) Cumulative fatalities assuming fixed age-specific case fatality rates (see *Materials and Methods*). (*D*–*F*) Same as *A*–*C*, but assuming that specific control measures are introduced when daily cases reached 10,000 (day 57): Older individuals social distance, and symptomatic individuals self-isolate (at 20% effectiveness). (*G*–*I*) Same as *D*–*F* but, in addition, schools close on day 57. Reopening of schools after 100 d results in a resurgence.

In the absence of any intervention measures, our modeling suggests SARS-CoV-2 will spread rapidly through the United Kingdom (Fig. 1*A*), and ultimately infect approximately 77% of the population (Fig. 1*B*). Using data on the age-specific fatality rate of COVID-19 (22), our results show around 350,000 fatalities among individuals aged over 60 y, and around 60,000 aged below 60 y (Fig. 1*C*). While we caution that our model makes a number of simplifying assumptions [e.g., no spatial dependence in transmission (11)], the total of fatalities is comparable to predictions made in other UK modeling studies (e.g., within the 95% prediction interval of ref. 20).

Sustained social distancing by older individuals (assumed to result in a 90% reduction in contacts with individuals under 25 y old, a 70% reduction with 25- to 59-y-olds, and a 50% reduction between one another), and moderately effective self-isolation by symptomatic individuals (at 20% efficacy) results in a shallower epidemic curve (Fig. 1*D*) and a much smaller outbreak size among individuals aged 60+ y (Fig. 1*E*). The attendant mortality burden among 60+-y-old individuals is also substantially reduced (to 62,000), with a smaller reduction in fatalities in those aged <60 y (to 43,000; Fig. 1*F*).

The addition of school (and university) closures, corresponding to a 70% reduction in contacts among school-aged individuals and a 20% reduction with 25- to 59-y-olds, dramatically reduces the rate of epidemic growth (Fig. 1*G*), although such levels of control are insufficient to suppress the epidemic (i.e., the number of daily cases still rises after implementation). The premature reopening of schools after 100 d (while the virus is still circulating) triggers a second wave of infection, with only a moderately reduced peak in daily new cases, largely eroding any additional gains made (23). The final proportion of the population exposed (Fig. 1*H*) and the number of fatalities (Fig. 1*I*) are largely unaltered compared to if schools had not been closed (compare Fig. 1 *E* and *F*).

**Fig. 2.**
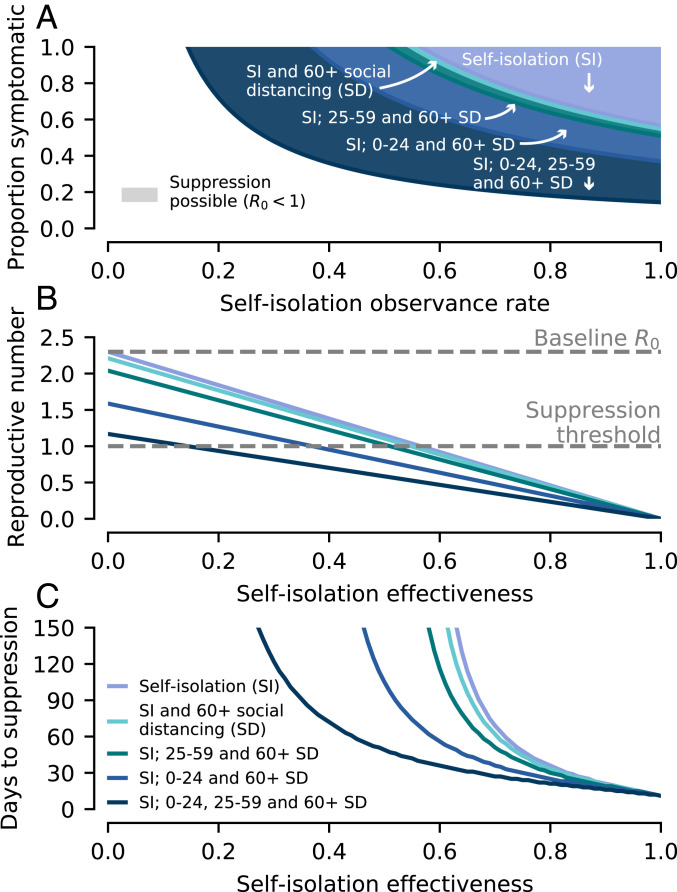
Prospects for disease suppression. Simulations of the age-structured SEIR model performed assuming control measures are initiated when there is a total of 10,000 infectious individuals in the population. The different control measures and strengths are listed in *Materials and Methods*. (*A*) Whether suppression is possible depends on both the self-isolation observance rate and the proportion of infections due to symptomatic individuals. More extensive social distancing measures increase the ranges of these two parameters for which suppression is possible. (*B*) Increases in self-isolation effectiveness drive down the reproductive number, which also depends on the social distancing measures employed. (*C*) The time taken for COVID-19 to be suppressed (modeled as a 100-fold reduction in infectious individuals) depends on the amount the reproductive number is decreased below one.

Our modeling indicates that, if sustained, such control measures can lead to the suppression of COVID-19 in the United Kingdom by reducing *R*_0_ to <1 (Fig. 2 *A* and *B*). The effectiveness of self-isolation by symptomatic individuals (i.e., its impact on reducing overall transmission) is a product of two factors. Firstly, the proportion of infections generated while the primary case is exhibiting symptoms (including mild symptoms) and, secondly, the engagement of symptomatic individuals with self-isolation policies (see *Materials and Methods*). As both of these parameters decrease, the self-isolation efficacy drops, and greater social distancing measures are necessary to achieve suppression (Fig. 2*A*). At present, there is a large uncertainty in the relationship between symptoms and viral shedding (9). For the social distancing strengths considered, suppression is possible if over 14% of infections are caused while the primary case is exhibiting symptoms ($p_{s}$). The associated self-isolation observance necessary to achieve suppression is inversely proportional to $p_{s}$, decreasing from 100% if $p_{s}=0.14$ to 50% if $p_{s}=0.28$. Given the uncertainty surrounding asymptomatic transmission, the likelihood of successful suppression is greatest if all social distancing measures are enacted (Fig. 2*B*).

The time taken for suppression to be achieved (modeled as a 100-fold reduction in infectious individuals) once control measures are implemented is shown in Fig. 2*C*. If self-isolation effectiveness is high (>70% reduction in transmission), then suppression can be achieved in 2 mo regardless of any additional social distancing measures. There is little additional decrease in the necessary duration of social distancing unless schools and workplaces are both closed, in which case suppression can be achieved within 2 mo at much lower levels of self-isolation effectiveness (≳45%).

**Fig. 3.**
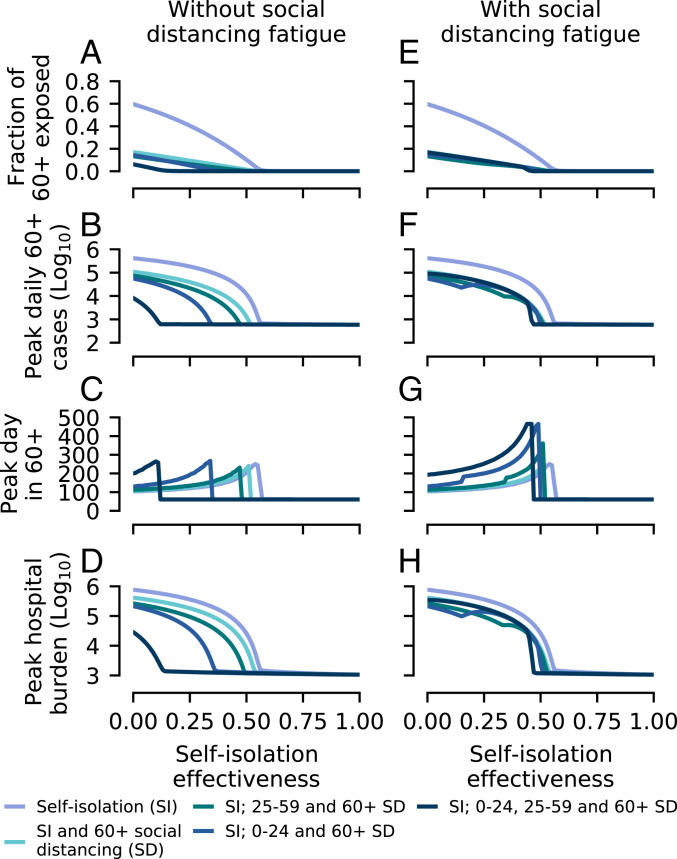
Outcomes of disease mitigation attempts, simulated using the age-structured SEIR model. As in Fig. 2, control measures are implemented when there are 10,000 cases in the population. (*A*) The final fraction of 60+-y-old individuals exposed to COVID-19 for each of the control strategies simulated, assuming that social distancing measures can be maintained at the same strength indefinitely (“without fatigue”). (*B*) Size and (*C*) timing of the peak in daily new cases among 60+-y-old individuals. (*D*) Peak hospital burden, assuming age-specific hospitalization rates (see *Materials and Methods*) and a mean hospital stay of 7 d (24). (*E*–*H*) Simulation results using the same control strategies as in *A*–*D*, but assuming that, due to fatigue, schools and workplaces closures last 100 d.

If suppression cannot be achieved (due to unfeasibility or lack of political will to reduce transmission sufficiently), then the objective of control measures is mitigation. Social distancing by 60+-y-old individuals results in a marked reduction of the final fraction of this age group that are exposed; however, unless both schools and workplaces are closed, additional social distancing measures do not lead to much further reduction (Fig. 3*A*).

These results are also mirrored in the impacts of social distancing on the daily cases in 60+-y-old individuals (Fig. 3*B*). Unfortunately, the hospital burden remains high for most intervention strategies, unless self-isolation is very effective (≳50%; Fig. 3*D*). Taking around 100,000 hospitalized cases to be the upper limit of hospital capacity, we find that there is a relatively small range of parameters where mitigation is successful at preventing hospitals being overwhelmed, but the disease is not also successfully suppressed (Fig. 3*D*; compare to Fig. 2*B*). If social distancing is applied to all age groups, this range is 0 to 14% self-isolation effectiveness, whereas, if just the 60+-y age group socially distance, the range is 41 to 54%.

As mentioned previously, if schools and workplaces reopen simultaneously after 100 d [e.g., due to social distancing “fatigue” (10)] and the disease has not been successfully suppressed, then much of the benefit of their closure is lost (Fig. 3 *E*–*H*) due to a resurgent second wave. In this scenario, the principle effect of school and workplace closures is in delaying the peak, buying more time for preparations (Fig. 3G).

It has been suggested that children might have reduced susceptibility to infection with SARS-CoV-2 (25). We repeated our analysis of suppression and mitigation assuming 50% reduction in susceptibility of individuals under 20 y old, with transmission rates among adults increased to ensure the same reproductive number, $R_{0}=2.3$. As might be expected, there is a decreased impact of school closures; however, this is compensated by the increased impact of social distancing by over-20-y-olds, resulting in little net change in our findings if both work and school closures occur (*SI Appendix*, Figs. S1 and S2).

**Fig. 4.**
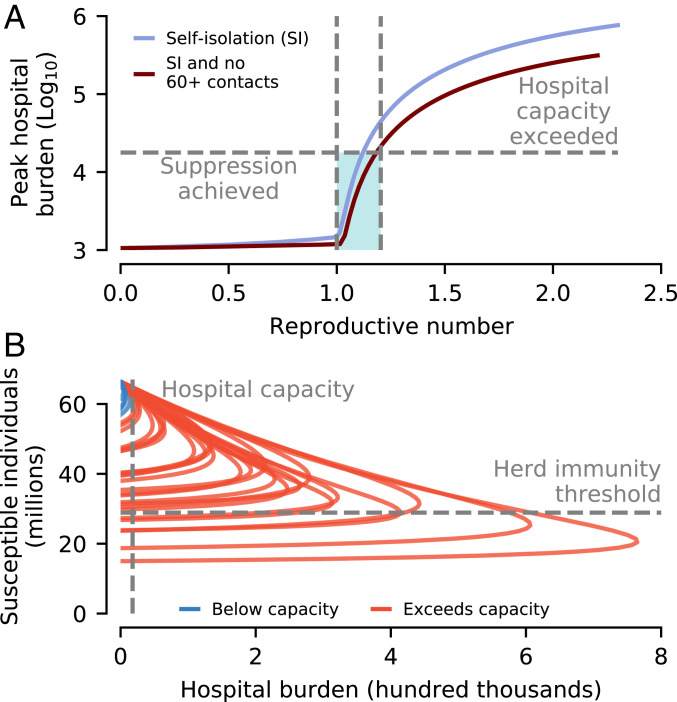
Summary of prospects for achieving herd immunity. (*A*) The peak hospital burden (shown on a log scale) is highly sensitive to the reproductive number. There is a narrow window of reproductive number values (shaded) where either 1) the number of COVID-19 cases requiring hospitalization does not overwhelm hospital capacity (modeled at the average hospital burden for April, 17,800 beds) or 2) circulation is suppressed. This window depends subtly on the exact age-specific social distancing configuration; however, all strategies studied fall between the two curves shown. (*B*) None of the simulated control scenarios shown in Figs. 2 and 3 achieved herd immunity while also keeping cases below hospital capacity. For the parameters considered, a hospital capacity in excess of 300,000 is required for this to be possible—almost 3 times the total UK NHS hospital beds (around 125,000 beds; see *Materials and Methods*), and around 15 times the average hospital burden of April.

To summarize, aiming to build herd immunity to SARS-CoV-2 in a population while mitigating the burden on hospitals requires initially reducing the reproductive number to ensure available hospital capacity is not exceeded (Fig. 4*A*). If social distancing is maintained at a fixed level, hospital capacity needs to be much larger than presently available to achieve herd immunity without exceeding capacity; otherwise, the final outbreak size will be insufficient to achieve herd immunity (Fig. 4*B*). Relaxing social distancing measures linearly is also insufficient (*SI Appendix*, Fig. S3).

**Fig. 5.**
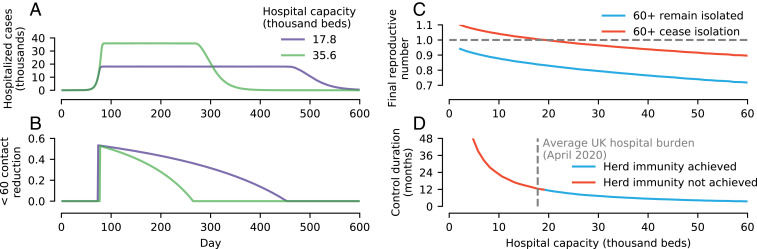
Proof-of-principle strategy for achieving herd immunity. This strategy 1) achieves herd immunity with minimal social distancing duration 2) without exceeding hospital capacity, and 3) prevents infection among the most vulnerable. We simulated a reduced two-age group (<60 y old and ≥60 y old) model, assuming that all ≥60-y-old individuals completely isolate. (*A*) To achieve herd immunity in the minimum time requires control measures that fix the rate of new hospitalized cases to ensure hospital beds are at the maximum acceptable capacity until herd immunity is achieved (results showing taking capacity to be 1× and 2× the average hospital burden of April). (*B*) The reduction in contacts (via the product of self-isolation and social distancing) among <60-y-olds needs to vary nonlinearly. Until the hospital burden hits the hospital capacity, there is no social distancing; subsequently, it is tuned to ensure $R_{e\mathrm{ff}}=1$; that is, the epidemic neither grows nor shrinks. Contact rates have to then gradually increase to exactly balance the reduction in $R_{e\mathrm{ff}}$ due to susceptible depletion. Eventually, there are no longer enough <60-y-old susceptible individuals to sustain the epidemic, and $R_{e\mathrm{ff}}<1$. (*C*) While this strategy ensures that the final reproductive number drops below 1 if individuals ≥60 y old remain in isolation, if they return to pre-COVID-19 contact rates, then whether the reduction in susceptibles is sufficient to achieve herd immunity depends on the hospital capacity. The greater the proportion of the population that needs protecting, the greater the hospital capacity needed to achieve herd immunity. (*D*) The duration of social distancing also depends on the available hospital capacity. For the parameters considered, if hospital capacity is less than around 20,000, then it is not possible to achieve herd immunity without individuals aged ≥60 y remaining socially distanced.

Instead, achieving herd immunity without exceeding hospital capacity requires that social distancing is implemented nonlinearly. An idealized optimal strategy for achieving social distancing, calculated using a two-age group (<60 y old and ≥60 y old) SEIR model, is shown in Fig. 5 *A* and *B*. We assumed that individuals ≥60 y old were able to perfectly self-isolate, while herd immunity is built up in the <60-y age group. The epidemic in <60-y-old individuals is initially allowed to grow unimpeded until the hospital burden caused by the number of sick individuals reaches the hospital capacity. At this point, social distancing among those younger than 60 y commences (Fig. 5*B*), at a level that ensures the epidemic growth stalls (i.e., $R_{e\mathrm{ff}}=1$). To achieve herd immunity in minimal time, the rate of new infections must then remain at the maximum afforded by the healthcare capacity. This requires reducing social distancing (i.e., increasing contacts) at the exact rate to balance out reductions in transmission stemming from the decreasing susceptible population (Fig. 5*B*)—too quickly and the epidemic grows above hospital capacity; too slowly and the epidemic with die out without reaching herd immunity. Over time, social distancing in <60-y-olds drops to zero, after which there are no longer enough susceptible <60-y-old individuals for the epidemic to be sustained. New infections continue but decline in frequency (meanwhile, further decreasing the effective reproductive number), before reaching zero.

While, at this point, the effective reproductive number is below 1 without any social distancing in the <60-y age group, full herd immunity has not necessarily been achieved (Fig. 5*C*). If ≥60-year-olds cease self-isolating, then the increased contact rates cause the effective reproductive number to increase, and may take it above 1. Achieving herd immunity requires that, while ≥60-y-olds are isolated, the additional new infections after $R_{e\mathrm{ff}}<1$ sufficiently further reduce $R_{e\mathrm{ff}}$ (the “overshoot”) such that, when ≥60-y-olds cease self-isolation, it remains below 1. The amount of overshoot increases with the hospital capacity (Fig. 5*C*). In addition to increasing the prospects of achieving herd immunity (and also its robustness; see also *SI Appendix*, Fig. S3*C*), the necessary duration of social distancing is inversely proportional to hospital capacity (Fig. 5*D*).

Our modeling required estimates for the case hospitalization probability (22) and mean hospitalization stay (24). We explored sensitivity of the duration of social distancing to these two parameters, and also performed a comparison with a rough calculation of the duration based on estimates of seroprevalance at the end of April (26) (*SI Appendix*, Fig. S4). Our estimated necessary social distancing duration of 12 mo is close to the estimate from serology of 7 mo to 11 mo, especially considering the uncertainty in the case hospitalization probability (22).

Throughout our analysis, we used $R_{0}=2.3$, broadly in line with most early estimates; however, some estimates were higher. We repeated our analysis using $R_{0}=5.7$ (27), finding that, as might be expected, the range of intervention parameters that led to successful suppression was reduced (*SI Appendix*, Fig. S5). However, our main finding was reinforced: Higher values of $R_{0}$ narrow the window for successful mitigation (*SI Appendix*, Fig. S6).

## Discussion

Various governments have entertained the idea of achieving herd immunity through natural infection as a means of ending the long-term threat of COVID-19. This has provoked alarm in sections of the public health community (16, 28). Our work confirms that this alarm is well founded.

Attempting to achieve herd immunity while simultaneously mitigating the impact of COVID-19 on hospital burden is an extremely challenging task. In order to ensure the hospital burden does not exceed levels comparable with that of the United Kingdom in April 2020, $R_{0}$ needs to be reduced from its initial value (assumed to be $R_{0}=2.3$) to about 1.2. Suppression is possible if $R_{0}$ is reduced below 1. Due to the fine margins (in terms of control effectiveness) between successful disease suppression and overwhelming hospitals, making herd immunity the primary objective (rather than applying maximal social distancing and aiming for suppression) is not supported by our modeling. Put another way, mitigation (via “flattening the curve”) is not a practical objective: If mitigation efforts are sufficient to prevent hospitals from being overwhelmed, only a comparatively small further increase in control measures will drive $R_{0}$ below 1, and make suppression possible.

If herd immunity is the objective, then, in addition to the narrow range of $R_{0}$ that must be aimed for, social distancing measures must be subsequently relaxed gradually in a highly controlled manner over a period of months to years. We were able to find a mathematical solution for building herd immunity with the minimal duration of social distancing using a reduced two-age group model. This solution requires knowledge of unobserved epidemiological determinants, namely, the remaining susceptible population, fraction exposed, and hospitalization probability. Additionally, complexities neglected by the model (e.g., failure of the ≥60-y age group to completely isolate, spatial structure) will alter the exact social distancing function. We, instead, view our results as serving as an indicator of the general shape and duration of social distancing necessary. Developing methods for implementable social distancing strategies capable of building toward herd immunity and that rely only on observable epidemiological data (e.g., the incidence curve and the hospital bed occupancy) requires further research.

The estimates of hospitalization probability and fatalities were calculated using results from a study of cases in Wuhan, China (22). For this study, we took the point estimates; however, these had uncertainties associated with them and are unlikely to be the same across regions. Furthermore, as treatment of COVID-19 continues to improve, fatality rates will fall. While there is an obvious feedback between fatality rates and healthcare system burden, the extent to which the Wuhan healthcare system (used in estimation) was overwhelmed is unclear. We therefore assumed the fatality rates fixed regardless of hospital burden. For these reasons, we have avoided attaching confidence intervals to estimates of fatalities, and they should be interpreted as plausible projections and not predictions.

A major unknown remains the nature, duration, and effectiveness of natural immunity. Here, we made the pragmatic assumption that, over the time scales under consideration, infection confers perfect long-lasting immunity (the best-case scenario for mitigation strategies). If immunity is not perfect, and there is a moderate to high chance of reinfection, then prospects for achieving herd immunity via natural infection are slim (29). To shed light on the kinetics of immunity, mass longitudinal antibody testing is necessary. This would both permit the identification of previously infected individuals, and provide information regarding immunity through time (30). We submit that models such as the one explored here, when integrated into statistical inference algorithms (31), provide a powerful means of integrating parallel serological and epidemiological data streams to quantify population-level immunity. Further, such models can be central to the development of efficient age-stratified serological testing schemes.

Finally, we stress that our study only explored the epidemiological impacts of nonpharmaceutical interventions (social distancing and self-isolation). Ultimately, any comprehensive public health policy needs to take into account the concomitant and wide-ranging societal and economic consequences of control measures.

## Materials and Methods

### Model.

We used a deterministic age-structured SEIR transmission model to simulate COVID-19 transmission in the United Kingdom. Contact rates $c_{i,j}$, the number of daily contacts an individual of age i y makes with individuals of age j y, were taken from the POLYMOD study of social mixing patterns for the United Kingdom (32) corrected for reciprocity (33). The simulated age groups were matched to those of the POLYMOD study: 14 5-y increments from 0 y to 69 y and then 70+ y. Age-stratified population sizes ($N_{j}$) were taken from 2018 UK demographic data.

The mean latent and infectious periods were set to 1/ρ=3 and 1/γ=3 d, respectively, consistent with various estimates of the serial interval (34, 35) and incubation period (4, 36, 37), assuming infectiousness starts 1 d to 2 d before symptoms develop.

Both latent and infectious periods were assumed to be gamma distributed and modeled using the method of stages (11, 38, 39), by dividing the exposed ($E_{i}$) and infectious ($I_{i}$) compartments for each age group into four subcompartments, $E_{i}=\sum_{k=1}^{4}E_{i}^{\left(k\right)}$ and $I_{i}=\sum_{k=1}^{4}I_{i}^{\left(k\right)}$, where the superscript labels the subcompartment. The transmission dynamics for the age groups were governed by a system of ordinary differential equations,

$$\frac{dS_{i}}{dt}=-\lambda_{i}\left(t\right)S_{i},$$ [1]

$$\frac{dE_{i}^{1}}{dt}=\lambda_{i}\left(t\right)S_{i}-4\rho E_{i}^{\left(1\right)},$$ [2]

$$\frac{dE_{i}^{k}}{dt}=4\rho E_{i}^{\left(k-1\right)}-4\rho E_{i}^{\left(k\right)}\text{for}k=2,3,4,$$ [3]

$$\frac{dI_{i}^{1}}{dt}=4\rho E_{i}^{\left(4\right)}-4\gamma I_{i}^{\left(1\right)},$$ [4]

$$\frac{dI_{i}^{k}}{dt}=4\gamma I_{i}^{\left(k-1\right)}-4\gamma I_{i}^{\left(k\right)}\text{for}k=2,3,4,$$ [5]

$$\lambda_{i}\left(t\right)=\beta \underset{j}{\sum }c_{i,j}\left(t\right)\frac{I_{j}\left(t\right)}{N_{j}}.$$ [6]

The transmission rate β was tuned using the next-generation matrix (40) to give a value of $R_{0}=2.3$, consistent with estimates (34, 35). Simulations were initialized with one initial introduction in a fully susceptible population ($S_{i}=N_{i}$). The resulting doubling time was observed to be about 3 d, broadly consistent with early observations from the United Kingdom.

### Modeling Nonpharmaceutical Interventions.

**Fig. 6.**
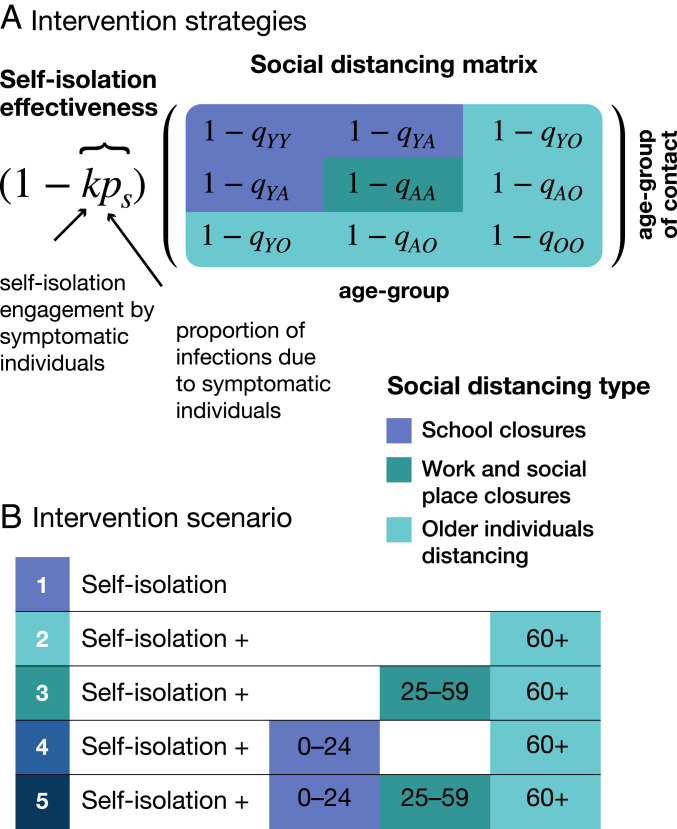
Modeling the impact of nonpharmaceutical interventions on disease transmission. (*A*) Different social distancing measures (e.g., school closures, work and social place closures, and older individuals distancing) reduce contact rates between individuals in different ways. To reduce the complexity of the model, and to understand their differential impacts, we assume that individuals are only affected by one of these measures, dependent on their age. Individuals aged 0 y to 24 y are affected by school closures (included in this are university closures). School closures were assumed to result in a 70% reduction in contacts among school-aged individuals ($q_{YY}$) and 20% reduction in their contacts with individuals aged 25 y to 59 y ($q_{YA}$). Work and social place closures were assumed to reduce contacts among adults ($q_{AA}$) by 50%. Finally, older individuals distancing reduced contacts by 60+-y-old individuals with 0- to 24-y-olds by 90% ($q_{YO}$), with 25- to 59-y-olds by 70% ($q_{AO}$), and among one another by 50% ($q_{OO}$). The effectiveness of symptomatic individuals self-isolating is dependent on two factors: 1) the observance by symptomatic individuals, k, and 2) the proportion of transmission due to individuals who are symptomatic, $p_{s}$. (*B*) We modeled five distinct combinations of social distancing measures, assuming that older individuals’ social distancing will always be prioritized.

Two types of nonpharmaceutical intervention were modeled: 1) self-isolation by symptomatic infectious individuals and 2) mass social distancing by differing age groups (Fig. 6). The effectiveness of self-isolation of symptomatic individuals is dependent on the product of two factors: 1) the proportion of infections that occur due to symptomatic individuals (excluding both presymptomatic and asymptomatic transmission), $p_{s}$, and 2) the observance rate of social isolation among symptomatic individuals, k. The fractional reduction of contacts between age groups i and j due to social distancing is given by $q_{i,j}$.

Both of these interventions take the form of modifications to the contact matrix between infectious and susceptible individuals,

$${\tilde{c}}_{i,j}=\left(1-kp_{s}\right)\left(1-q_{i,j}\right)c_{i,j}.$$ [7]

This expression for ${\tilde{c}}_{i,j}$ is inserted in place of $c_{i,j}$ in Eq. **6**.

Age groups in the model are divided into whether they are young (Y; corresponding to 0- to 24-y-olds and age groups i=1 to 4), adults (A; 25- to 59-y-olds, age groups i=5 to 11) and older (O; 60+-y-olds, age groups i=12 to 15). The reduction in contacts due to social distancing, $q_{i,j}$, is then determined by which of these three categories the contacter and contactee fall into, given by the block matrix

$$q=\left(\begin{array}{ccc} q_{Y\;Y} & q_{Y\;A} & q_{Y\;O}\\ q_{Y\;A} & q_{AA} & q_{AO}\\ q_{Y\;O} & q_{AO} & q_{OO} \end{array}\right).$$ [8]

We assume school closures reduce contact rates between young individuals by a factor of $q_{Y\;Y}=0.7$ and between young people and adults by $q_{Y\;A}=0.2$. Social distancing among adults (e.g., due to workplace closures and reduction in social events) was modeled as a reduction of $q_{AA}=0.5$. Social distancing of older individuals was represented by $q_{Y\;O}=0.9$, $q_{AO}=0.7$, and $q_{OO}=0.5$. For simulations with social distancing fatigue, $q_{Y\;Y}$, $q_{Y\;A}$, and $q_{AA}$ were modeled as linearly decreasing from these initial values to 0 over the periods indicated in *SI Appendix*, Fig. S3.

### Estimating Hospital Burden and Case Fatalities.

Age-specific hospitalization probabilities, $h_{i}$, and fatality rates were taken from point estimates calculated in a study of cases in Wuhan, China (22). Due to differences in the final age group of our model (70+ y) and those of the Wuhan study (70- to 79-y-olds and 80+-y-olds), the hospitalization and fatality rates for 70+-y-old individuals were calculated by summing the estimated 70- to 79-y-old and 80+-y-old rates weighted by their relative UK population sizes. Based on data from the United Kingdom, we assumed the average duration of hospitalization with COVID-19, d, was 7 d (24).

Pre–COVID-19 hospital beds and occupancy rates in the UK National Health Systems (NHS) were taken from the most recent (autumn 2019) published numbers for Northern Ireland, Wales, Scotland, and England.

Hospital burden was calculated as $H=d\sum_{i}h_{i}\Lambda_{i}S_{i}$. Although there is a lag between exposure and hospitalization, this has no bearing on our mathematical results.

### Optimal Strategy to Achieve Herd Immunity.

We used a two-age group model (<60-y-olds and ≥60-y-olds) to demonstrate an optimal social distancing strategy while shielding ≥60-y-old individuals. To preserve the data-informed contact structure, we made the approximation that the age-specific incidence was constant for age groups <60 y and similarly for ≥60 y. The two-age group contact matrix is then given by a weighted sum of the POLYMOD-derived contact matrix, $c_{a,b}^{\left(2\right)}=\sum_{i\in a}\sum_{j\in b}\left(N_{i}/N_{b}\right)c_{i,j}$, where a and b index age groups {<60,≥60}. Aside from the contact matrix, the model is identical in structure to that given in Eqs. **1**–**6**.

If ≥60-y-old individuals are shielded (contact rates reduced to zero), the <60-y-old susceptible population is depleted at the fastest rate if the epidemic is allowed to grow unimpeded until the hospital burden (as defined above) reaches the hospital capacity, $H_{c}$. At this point, contact rates are reduced to maintain hospital burden at hospital capacity. Mathematically, this translates into setting the reduction in contacts, q(t), such that Eqs. **2**–**5** all equal 0. Solving for q(t) gives

$$q\left(t\right)=\left\{\begin{array}{cc} 1-1/R_{e\mathrm{ff}}^{u}\left(t\right) & \text{if}H\geq H_{c}\text{and}R_{e\mathrm{ff}}^{u}\left(t\right)>1,\\ 0 & \text{otherwise}. \end{array}\right.$$ [9]

where $R_{e\mathrm{ff}}^{u}\left(t\right)=\gamma N_{0}/\left(\beta c_{0,0}^{\left(2\right)}S_{0}\left(t\right)\right)$ is the effective reproductive number without contact reduction by <60-y-olds. Substituting Eq. **9** into Eq. **1** and solving gives an expression for the control duration, $t_{d}$. For the parameters considered, there is limited susceptible depletion prior to hospital capacity being reached, and so

$$t_{d}\approx \frac{dh_{0}N_{0}}{H_{c}}\left(1-\frac{\gamma }{\beta c_{0,0}^{\left(2\right)}}\right).$$ [10]

## Supplementary Material

Supplementary File

## Data Availability

Source code data have been deposited in Zenodo (10.5281/zenodo.400093) (41).
