# Supplementary material for: Transmission dynamics reveal the impracticality of COVID-19 herd immunity strategies
Source: Proc Natl Acad Sci U S A. 2020 Sep 22;117(41):25897–903. doi: 10.1073/pnas.2008087117 (PMC7568326; doi:10.1073/pnas.2008087117)
Supplement: Supplementary File [file pnas.2008087117.sapp.pdf]

PNAS
www.pnas.org

Supplementary Information for

Transmission dynamics reveal the impracticality of COVID-19 herd immunity strategies

Tobias S Brett, Pejman Rohani

Tobias Brett.
E-mail: tsbrett@uga.edu

This PDF file includes:

Figs. S1 to S6
SI References

www.pnas.org/cgi/doi/10.1073/pnas.2008087117

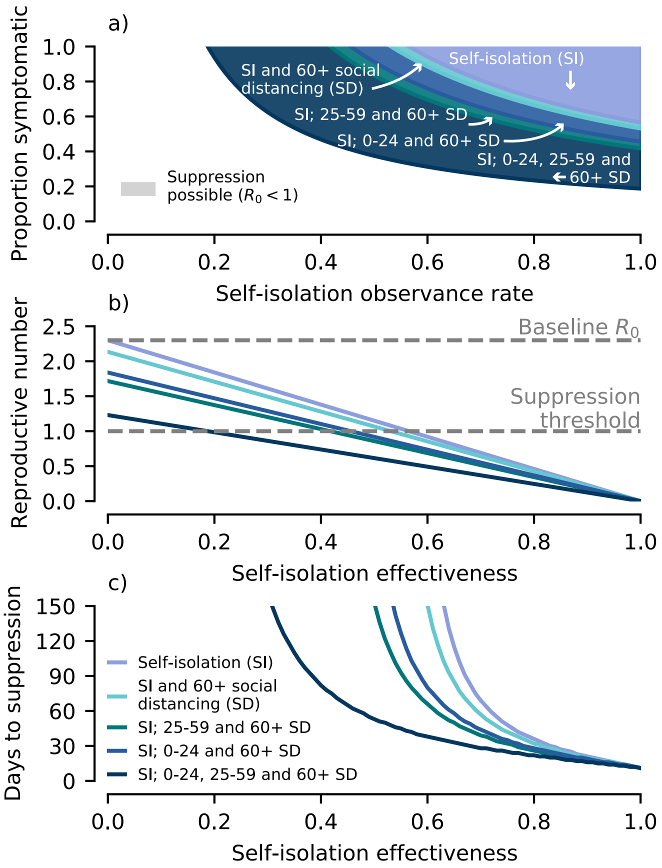

Fig. S1. Repeat of Fig. 2 (prospects for disease suppression) assuming that individuals under 20 have a 50% reduction in susceptibility to infection relative to older individuals. The transmission rate, β, was recalculated to ensure $R_0 = 2.3$.

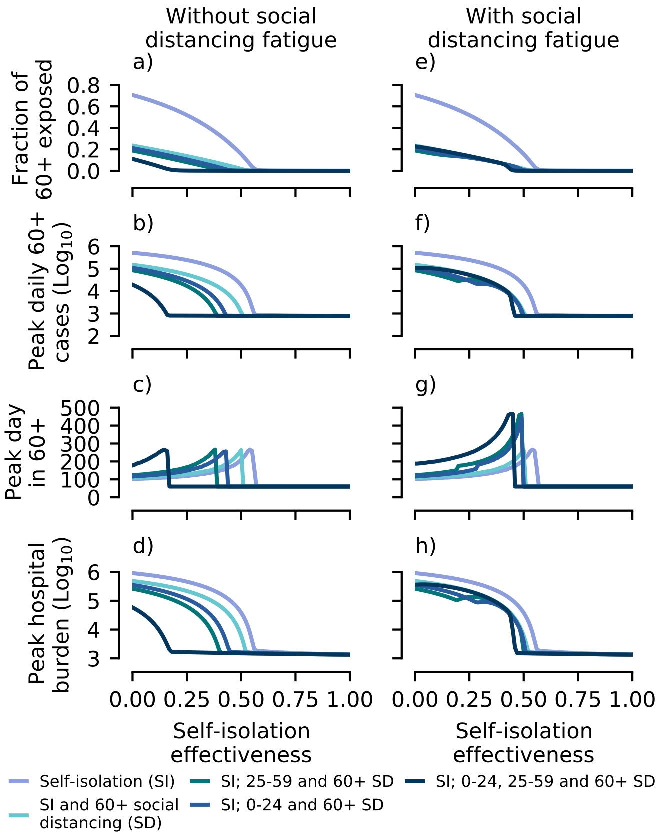

Fig. S2. Repeat of Fig. 3 (outcomes of disease mitigation attempts) assuming that individuals under 20 have a 50% reduction in susceptibility to infection relative to older individuals.

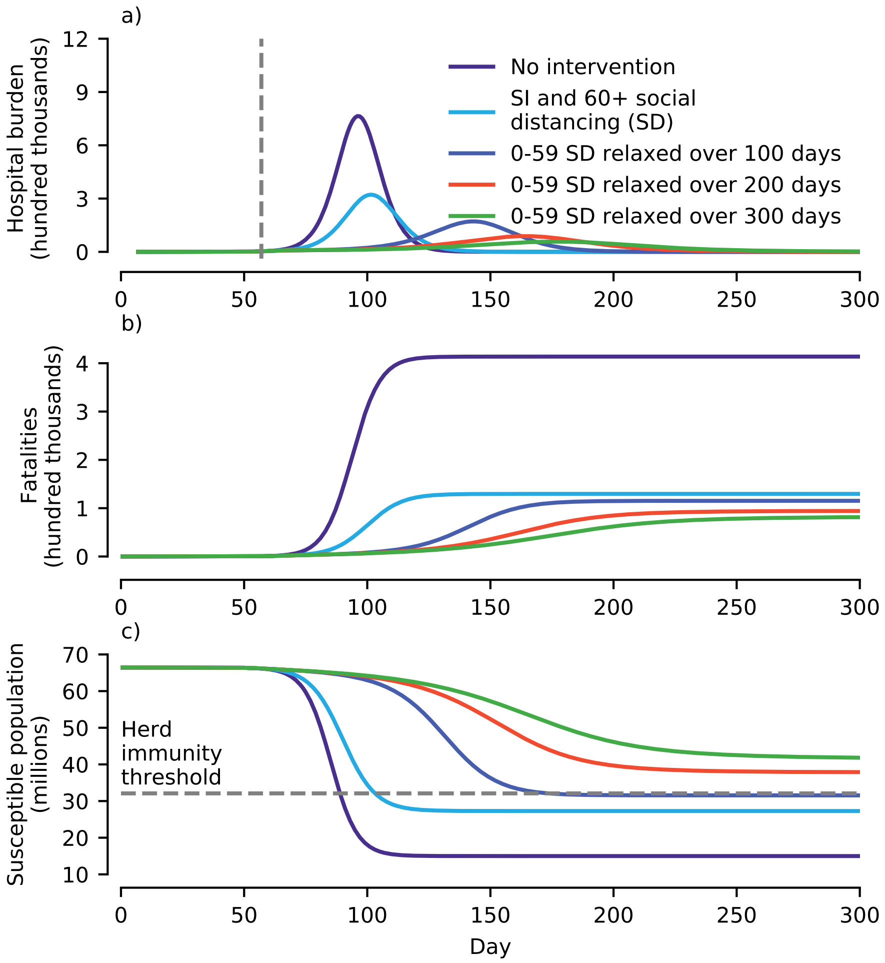

Fig. S3. Disease mitigation in the UK with gradual relaxation of controls. Simulated results generated using the age-structured SEIR model. For simulations with social distancing fatigue, q_{YY}, q_{YA} and q_{AA} were modelled as linearly decreasing from their initial values (see Methods) to 0 over the periods indicated. a) Inclusion of social distancing by 0-59 year-olds (e.g. due to school and workplace closures) reduces peak hospital burden. The more gradually social distancing by these age groups is relaxed, the greater the reduction. b) More gradual relaxation in controls results in a slightly smaller final number of fatalities. c) The overall reduction in the susceptible population below the herd immunity threshold is less the longer controls are applied. If social distancing is relaxed too slowly then the disease is eliminated before herd immunity is achieved, and the population remains vulnerable to reintroduction of the pathogen (as with suppression strategies). Simulated results assumed control measures are initiated when there is a total of 10 thousand infectious individuals in the population and that social distancing measures affecting 0-59 year-olds (school and workplace closures) are gradually relaxed. For all results shown (apart from where there was no intervention) self-isolation effectiveness was assumed to be maintained at 10%. Similarly, 60+ aged individuals were assumed not to relax their social distancing.

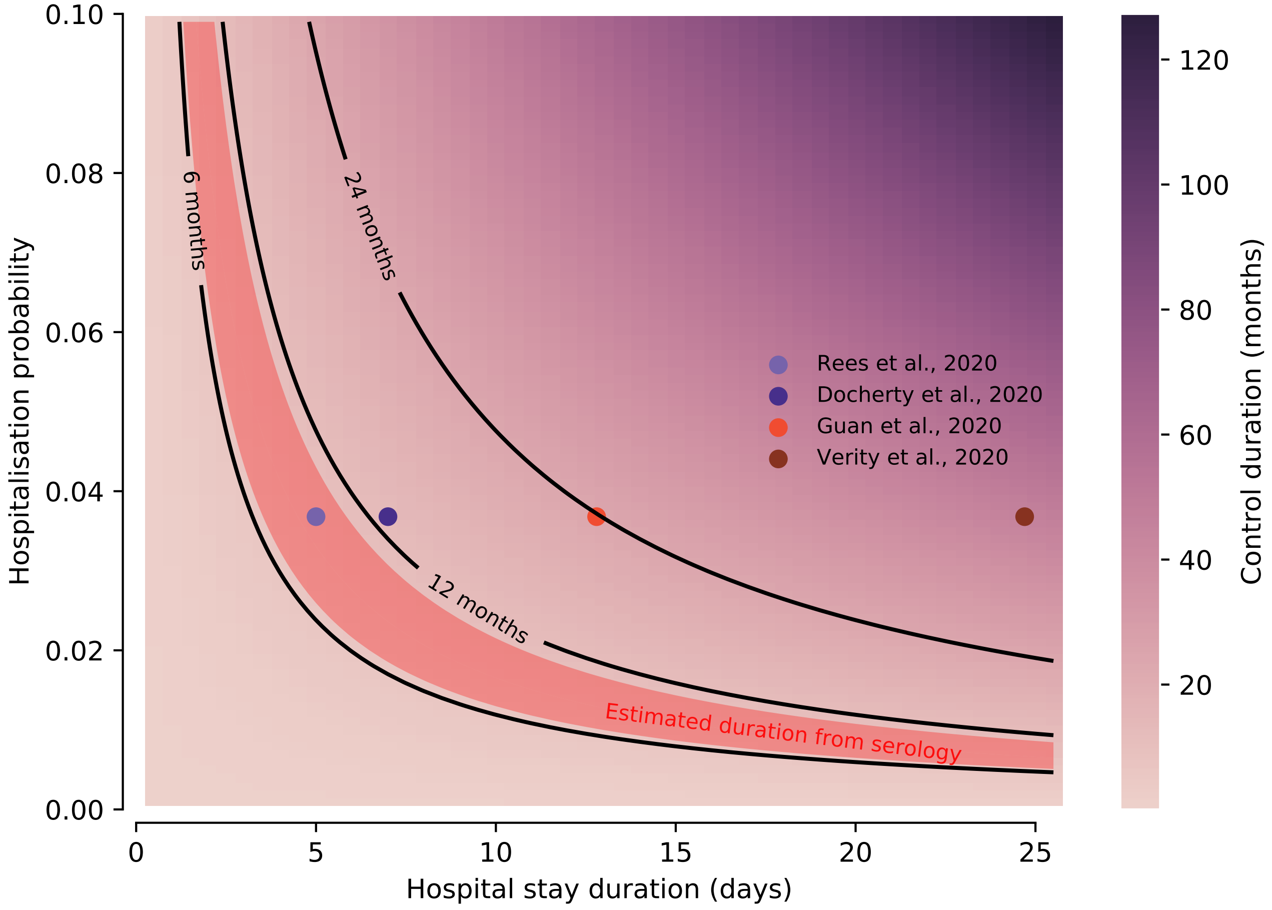

Fig. S4. Heat map showing the dependence of control measure duration on case hospitalisation probability and the mean hospital stay using the optimal control strategy. The control measure duration is calculated using Eq. 10 with β set to ensure $R_0 = 2.3$. The hospital burden was assumed to be maintained at 17800 beds occupied with COVID-19 patients, the UK average for the peak month of April 2020. There is uncertainty both in the hospitalisation probability and hospital stay duration of COVID-19 cases. Four estimates are shown, one from a meta-analysis (1), one from a study of UK patients (2), and two from separate studies of patients in Wuhan, China (3, 4). The hospitalisation probability, $h_p = 0.0368$ comes from the age-specific point estimates given in (4), weighted by UK demographics. Due to the inference procedure used in (4), we were not able to propagate the uncertainty, however, for context, the estimate for the 30-39 age-group hospitalisation probability was 0.0343, 95% credible interval [0.0204-0.0700]. As a comparison, we calculated a crude estimate of the necessary duration of control measures by extrapolating from serosurvey data. The seroprevalence of SARS-CoV-2 antibodies in the UK population at the end of April was estimated at 6.78%, 95% confidence interval [5.2, 8.64] (5). Assuming the herd immunity threshold is $h_I = 1 - 1/R_0$ (6), that hospital burden is maintained at April levels and that $R_0 = 2.3$, these seroprevalence estimates translate into a control duration of around 7 to 11 months (shown in the shaded coral region).

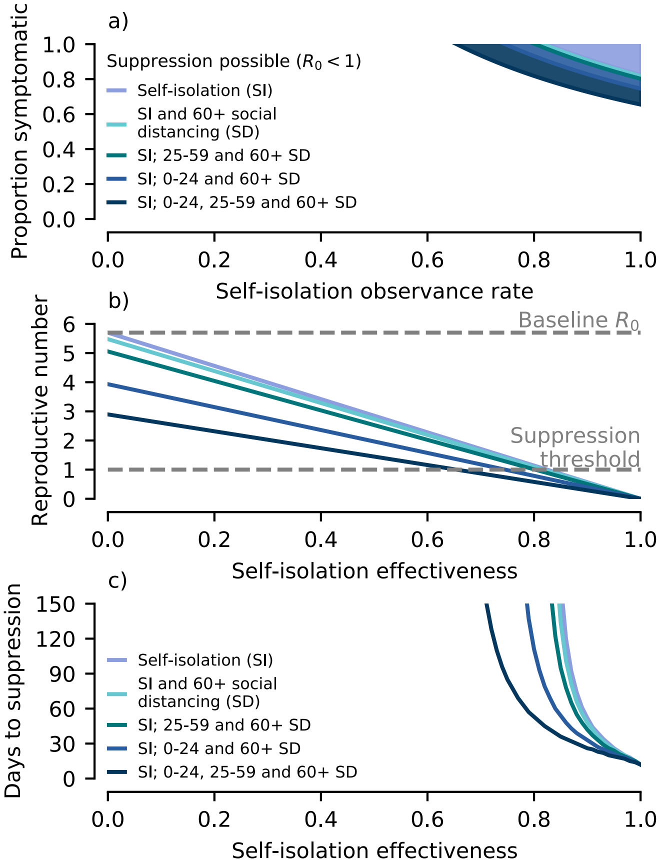

Fig. S5. Repeat of Fig. 2 (prospects for disease suppression) using $R_0 = 5.7$.

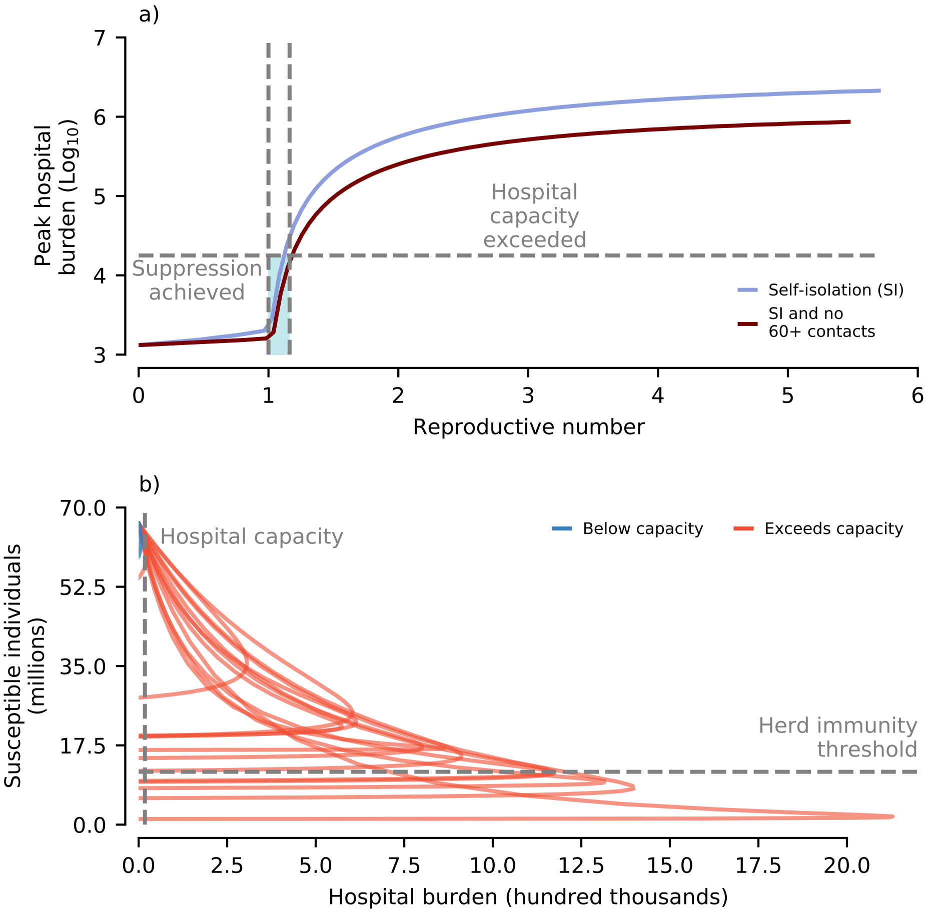

Fig. S6. Repeat of Fig. 4 (summary of prospects for achieving herd immunity) using $R_0 = 5.7$.

References

1. EM Rees, et al., COVID-19 length of hospital stay: a systematic review and data synthesis. *medRxiv* (2020).
2. AB Docherty, et al., Features of 16,749 hospitalised UK patients with COVID-19 using the ISARIC WHO Clinical Characterisation Protocol. *medRxiv* (2020).
3. WJ Guan, et al., Clinical characteristics of coronavirus disease 2019 in China. *New Engl. J. Medicine* (2020).
4. R Verity, et al., Estimates of the severity of coronavirus disease 2019: a model-based analysis. *The Lancet Infect. Dis.* (2020).
5. Office for National Statistics, Coronavirus (COVID-19) Infection Survey pilot: 28 May 2020. *Off. for Natl. Stat. Bull.* (2020).
6. MJ Keeling, P Rohani, *Modeling Infectious Diseases in Humans and Animals.* (Princeton University Press), (2008).
